# Social Capital and Self-Rated Health: A Cross-Sectional Study among Rural Japanese Working Residents

**DOI:** 10.3390/ijerph192114018

**Published:** 2022-10-27

**Authors:** Elijah Deku-Mwin Kuurdor, Hirokazu Tanaka, Takumi Kitajima, Jennifer Xolali Amexo, Shigeru Sokejima

**Affiliations:** 1 Department of Public Health and Occupational Medicine, Mie University Graduate School of Medicine, 2-174 Edobashi, Tsu-shi 514-8507, Japan; 2 Division of Surveillance and Policy Evaluation, Institute for Cancer Control, National Cancer Center, 5-1-1, Tsukuji, Chuo-ku, Tokyo 104-0045, Japan; 3 Department of Clinical Research, National Hospital Organization, Mie National Hospital, Tsu-shi 514-0125, Japan; 4 Epidemiology Centre for Disease Control and Prevention, Mie University Hospital, Tsu-shi 514-8507, Japan

**Keywords:** social capital, self-rated health, employment types, workers, Japan

## Abstract

Social capital is positively associated with self-rated health; however, this association among workers is still unclear. Thus, this study examined the relationship between social capital and self-rated health with special attention to the employment type. A cross-sectional survey was conducted with 6160 workers aged 20–64 years from two towns in Mie Prefecture in January–March 2013. Social capital was assessed using five items in 4816 income-earning workers. The social capital scores were summed and then divided into three groups. The self-rated health responses were dichotomised into ‘poor’ and ‘good’. The association was examined using a stepwise binomial logistic regression stratified by employment type and adjusted for potential confounders. Regular employees with low social capital had a higher significant odds ratio of poor self-rated health than medium (OR 0.58 95% CIs 0.39–0.87) and high (OR 0.39; 95% CIs 0.26–0.59) social capital levels after controlling for all potential confounders. Similar patterns were observed for non-regular employees with medium and high social capital. There was a significant relationship between some indicators of social capital and poor self-rated health among self-employees. These results highlight that social capital acts as an unequal health resource for different types of workers.

## 1. Introduction

The proportion of non-regular employees continues to rise, according to recent changes in the employment structure. Non-regular employees account for 38.7% of all Japanese employees, according to a recent survey performed by the Ministry of Health, Labour and Welfare in 2010 [1]. Simultaneously, there was considerable growth in the number of women and the elderly entering the labour force or continuing to work after retirement in recent years, mostly as non-regular employees [1]. The widening discrepancy between non-regular employees and other employment kinds (regular and self-employed) has become a social issue in these circumstances.

The impact of labour market structure trends on individual health outcomes can be both good and harmful. Changes in a person’s living environment and lifestyle are all linked to the type of work they do. Studies have shown that trust, neighbourhood interactions, and civic engagements are dwindling [2,3,4,5]. On the one hand, regular employees’ long working hours tend to affect social interaction time with neighbours, and inflexible employment conditions affect their lifestyle [6]. They, on the other hand, have a higher level of job security than both non-regular and self-employed workers [7]. Access to various community resources, such as health information, is impacted by these circumstances. The impact of the labour market structure on health is multifaceted, and it is driven purely by changes in reciprocity and trust, as well as by the social system and connections, which are all encompassed by most indicators of social capital.

Social capital is recognized as a valuable social resource with individual and communal characteristics that can be quantified and evaluated within a social network to achieve health and well-being benefits [8]. Through the use of social factors to explain community health, social capital as a product of the social environment has recently gained recognition as very crucial in public health. There is empirical evidence demonstrating the importance of social capital in health. A community with high social capital, for example, promotes healthier behaviours through the easy dissemination of information on health-promoting behaviours [9]. Similarly, members of a community with high social capital may have a stronger sense of mutual responsibility and informal social control to adhere to disease-prevention protocols [10,11,12]. After controlling for race, ethnicity, and socioeconomic factors, it was found to be negatively linked with mortality in other studies [13]. Individuals with higher levels of social trust, higher levels of associational involvement, more participation in organized interactions, more informal socializing, or those who volunteer, regarded themselves to be healthier than those with lower levels of these individual social capital indicators [14].

Self-rated health is a subjective measure of an individual’s perception of their health. This is a significant and independent health status indicator across different social demographic groups [15,16,17,18]. Different factors play a role in determining the self-rated health of an individual. In the case of workers, psychosocial work stressors are one of the most studied occupational factors, with nearly all studies examining one or more stressors in this area. Some studies have found temporary employment, social support job insecurity, and work-family balance to be associated with self-rated health [19,20,21], However, previous studies present conflicting findings regarding the relationship between the different employment types and self-rated health. For example, it has been reported that non-regular and self-employees are likely to have poorer self-rated health, while on the contrary, other studies report that among the working adults in the US, self-employed individuals are as healthy as regular employees (wage earners) [22,23]. To explain these opposing views, existing literature has suggested a few factors. Among the many factors cited for this disparity, such as gender, employment flexibility, and social support, social capital is one of the least studied [24].

Given the differences in employment stability, working hours, and flexibility among the various employment kinds, it is crucial to look at any potential relationships between social capital and self-rated health. This can lead to the adoption of a more targeted strategy directed at a particular employment type rather than a broad and generalised intervention based on social capital. Thus, this study seeks to explore the relationship between social capital and self-rated health with special attention to the employment type.

## 2. Materials and Methods

### 2.1. Data Collection

**Figure 1:**
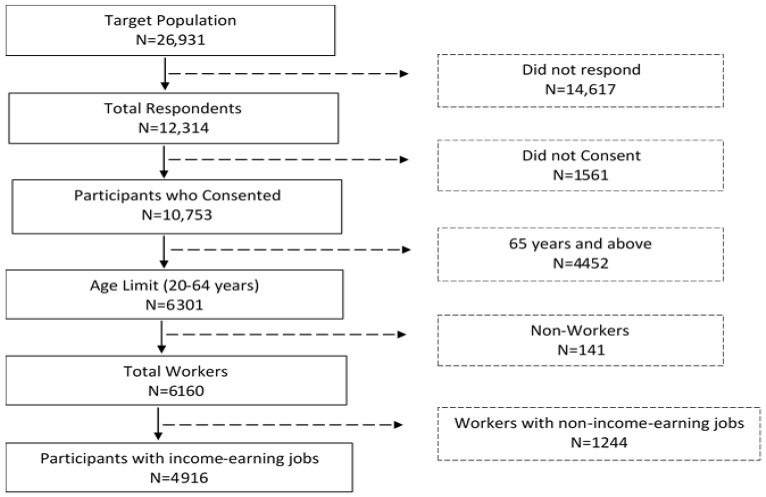
Sequential exclusion of participants in the study.

This empirical analysis employed population-based cross-sectional survey data obtained from two rural towns located in the eastern part of Mie Prefecture. Self-completion questionnaires were administered to 26,931 residents aged ≥20 on various topics (social capital, socioeconomic status, lifestyle, non-communicable diseases (NCDs), and self-rated health) in 2013 from January to March. A total of 10,753 of the 12,314 respondents consented to their information to be used in this study, representing an 87.3% valid participation rate. A total of 6301 residents were aged 20–64 years at the time of the survey and 6160 of the participants were workers. Of the total workers, 4916 of them had “income-earning jobs”. The respondents who did not answer the employment status question and did not have an income-generating job were excluded from the analysis. Figure 1 shows the sequential exclusion of participants in the study.

### 2.2. Measurement of Social Capital

The social capital questionnaire was drawn from the questionnaires from the Cabinet Office, Government of Japan (COJ), 2003 [25]. Individual participants’ understandings of social capital were used to evaluate social capital based on these five indicators: feeling of fellowship, social support, norms of reciprocity, perceived neighbourhood trust, and social activity or co-operation. To assess each of the measures, the following five items were asked: (1) I feel myself as a member of neighbours (feeling of fellowship); (2) I have someone in my neighbourhood to consult when I need to talk to someone (social support); (3) I like someone in my neighbourhood who has the same ideas as me (reciprocity norm); (4) I think people in my neighbourhood trust each other (perceived neighbourhood trust); (5) My neighbours and I perform social activities together for the betterment of the neighbourhood (social activity or co-operation) [26,27,28]. On a five-point Likert scale, these five items were scored as follows: strongly disagree, disagree, neither, agree, and strongly agree. The overall social capital score was used to categorize the participants into three groups based on the ratings of each item on the questionnaire. The first group, second group, and third group were defined as the ‘low’, ‘medium’, and ‘high’ social capital, respectively.

### 2.3. Self-Rated Health

The self-rated health variable, which is how respondents perceive their general health, was collected using a single questionnaire. Respondents were asked the following: “Generally, how was your health during the past month?” The responses were on a scale of six levels, including “excellent”, “very good”, “good”, “not so good”, “not good”, and “not good at all”. The answers were dichotomised to “poor health” = 1 (“not so good”, “not good”, “not good at all”) and “good health” = 0 (“excellent”, “very good”, “good”, “not so good”), as was done in other studies [29,30].

### 2.4. Covariates

Potential confounders were controlled for in the study. The following variables were included: gender; age (20–24, 25–34, 35–44, 45–54 and 55–64); annual household income (<JPY 2 million, JPY 2 million to less than 4 million, JPY 4 million to less than 6 million, JPY 6 million to less than 8 million, and JPY ≥8 million); and occupation (blue-collar, white-collar, and others) [27]. In addition, educational level was grouped into the following three categories: high (university, Master’s degree, and above), medium (college and vocational schools), and low (elementary school, junior, and senior high school). The marital status variable includes three categories: never married, currently married, and previously married.

Health-related characteristics of participants included smoking (currently smokes, do not smoke). For physical activity, according to the WHO recommendation for adults, physical activity of less than 150 min per week was considered physically inactive and greater than or equal to 150 min per week as physically active [31].

Based on WHO recommendations, Body Mass Index (BMI) was categorized as follows: underweight (<18.50), normal (18.50–24.99), overweight (25.00–29.99), and obese (≥30) [31]. Participants were categorized as having an NCD if they confirmed ever receiving a diagnosis of one or more of the following diseases: asthma, stroke, diabetes mellitus, myocardial infarction, allergy, hypertension, and cancer. Employment type was classified into three types: regular employee, non-regular employee (part-time, contract, or temporary worker), and self-employee.

### 2.5. Data Analysis

SPSS 26.0 software (SPSS Inc., Chicago, IL USA) was used for the statistical analysis. Descriptive statistics were used to describe the frequency and percentage of study sample variables. The relationship between the study population characteristics and social capital, based on employment type, within the groups was examined using chi-square (χ^2^). For missing data analysis, a multiple imputation method was employed to replace the missing values by using employment type. The variables in the imputation model were self-rated health, social capital, education, age, occupation, annual household income, marital status, smoking, BMI, physical activity, and NCD. Using stepwise binary logistic regression analysis, the prevalence odds ratio for the association between social capital and self-rated health was examined after controlling for covariates. It was started with a crude model and Model 1^a^ adjusted for the effects of gender and age. Model 2^b^ additionally adjusted for the effects of annual household income, education, marital status, and occupation. A final analysis was conducted with Model 3^c^ by further adjusting for smoking status, physical activeness, non-communicable disease, and BMI. The adjusted prevalence odds ratios and 95% confidence interval (CI) were calculated. The variance inflation factor (VIF) and tolerance value (TOL) were used to assess the degree of correlation among the independent variables. Furthermore, a reliability test was carried out to confirm this classification [32].

### 2.6. Ethical Considerations

Participation in the study was entirely voluntary and anonymous, and all individuals provided written informed consent. The Ethical Committee of the Medical Department of Mie University approved the study protocol (Approved number: 1268). Data collection was done under the principles of the Declaration of Helsinki.

## 3. Results

### 3.1. Demographic Characteristics of Respondents

The study population comprised 4916 participants. Table 1 shows the socio-demographic and self-rated health stratified by the employment type. The three groups: regular employees, non-regular employees, and self-employees, represented 52.4%, 33.4%, and 14.3%, respectively, of the study population. The larger proportions of each employment type were males, except for non-regular employees. Most regular employees were in the 45–54 age group, but both non-regular employees and the self-employed had the majority of members in the 55–64 age group. For the remaining socio-demographic characteristics, non-regular workers were more likely to have less education, have more white-collar jobs, and have a larger proportion with lower annual household incomes than both regular workers and the self-employed. Most participants were married, with the highest percentage being self-employed. The regular employee cohort had more workers who currently smoked when compared with both non-regular employees and self-employed workers. For the physical activity, presence of NCDs and self-rated health, there was no statistical difference among the three groups, though a significant difference was observed for BMI.

### 3.2. Distribution of Respondents among the Social Capital Levels

Table 2 shows the characteristic distribution of respondents among the social capital levels based on the employment type. According to the chi-square test, social capital levels were different for each employment type among the various characteristics. Social capital levels of non-regular employees were significantly different for all the characteristics except for gender, educational level, occupation, physical activity, and NCD. However, among self-employed workers, there were no significant differences among the social capital levels for respondent characteristics, such as self-rated health (*p* = 0.124), gender (*p* = 0.202), educational attainment (*p* = 0.54), occupation (*p* = 0.598), annual household income (*p* = 0.32), physical activity (*p* = 0.36), smoking status (*p* = 0.097), and marital status (*p* = 0.237). Similarly, social levels among non-regular employees were not significantly different for gender (*p* = 0.611), occupation (*p* = 0.374), physical activity (*p* = 0.213), and NCD (*p* = 0.709). Education level was the only characteristic that showed a significant difference among the social levels group of regular employees (*p* = 0.104). In both regular employees and non-regular employees, the social capital levels distribution of variables such as age, annual household income, marital status, smoking status, BMI, and self-rated health status differed significantly.

### 3.3. Prevalence Ratios of Social Capital for Poor Self-Rated Health Respondents

Table 3 provides the results of the stepwise binomial logistic regression using the multiple imputation method. Model 1^a^ controlled for age and gender and Model 2^b^ adjusted for the socio-economic factors, besides the age and gender of the participants. Finally, Model 3^c^ controlled for health-related behaviours and health status. Across all the models, significant associations were found between social capital at all levels and self-related health for both regular and non-regular employees. On the contrary, among the self-employed workers, there was no significant relationship across all the models. It can be observed that regular employees with low social capital levels had a significantly higher prevalence of poor self-rated health than medium (OR 0.583; 95% CIs 0.391–0.870) and high (OR 0.393; 95% CIs 0.262–0.590) social capital levels after controlling for all potential confounders. Similar patterns were observed for non-regular employees with medium (OR 0.466; 95% CIs 0.289–0.751) and high (OR 0.339; 95% CIs 0.209–0.550) social capital. There was no significant relationship between the social capital of self-employed workers and poor self-rated health.

### 3.4. Prevalence Ratios of Social Capital Dimensions for Poor Self-Rated Health Respondents

The analysis of the association between each indicator of social capital and self-rated health is shown in Table 4. There was a significant relationship among individuals with high levels of each indicator across all three types of employment except self-employees. Statistically, a significant association between high levels of feelings of fellowship, cooperation, social activities, and social support, and poor self-rated health were identified after adjusting for potential confounders among self-employees.

## 4. Discussion

Significant associations between social capital and self-rated health were found in this study among regular and non-regular employees, but not among self-employed workers. After adjusting for all potential confounders, social capital was found to be negatively associated with the prevalence of poor self-rated health. Furthermore, among regular and non-regular employees, each indicator of social capital was negatively associated with the prevalence of poor self-rated health. However, only the feelings of fellowship, social support, and cooperation were significantly related to self-rated health in self-employed people.

The benefits of social capital may not be reaped equally across various groups because of their unique environment. Long working hours, for example, imply less time spent with family and friends, with whom a greater proportion of social capital is accumulated. According to studies, poor working conditions and increased occupational stress are significant predictors of poor self-rated health [33,34,35]. The combination of these factors is expected to result in poor self-rated health. Contrary to this notion, the results show that respondents with high social capital among the non-regular employees are about 60% less likely to perceive their health as poor compared to those with low social capital. It further shows that high social capital can have a buffering function aimed at negating the effects of work-related stress. These benefits have also been observed empirically in different populations [36].

The results showed no association between social capital and poor self-rated health among the self-employed, even after controlling for the demographic and health-related behaviours. However, self-employees showed better self-rated health as compared with both regular and non-regular employees. They are known to have job autonomy, job control, and control over the length of working hours. This acts as an advantage to building high social capital. As shown in the results, they have the highest proportion of workers with high social capital. Their employment type may not be an impediment to the accumulation of high social capital with family and neighbours as those of the other employment types because they are physically closer and there is greater job autonomy [22]. Family and friends are major sources of social capital for this group of employees, and it is usually manifested in their feeling of fellowship and social support [37,38]. In effect, their social capital may not be a significant predictor of their poor self-rated health. Further research may be needed to elucidate this relationship.

Non-regular employees with high social capital had improved self-rated health than those with low social capital. All of the social capital indicators examined in this study were significantly related to self-rated health among non-regular employees. This is not surprising given the drawbacks of non-regular employment, including low pay, job insecurity, and inadequate social security [39]. This has implications for the self-perceived health and psychological health of employees. It was affirmed in research conducted among young Japanese researchers. It shows that employment insecurity and a perceived threat to employment continuity and stability mediated the relationship between non-regular employment and psychological distress that leads to poor mental health [40]. Among the measures of social capital, perceived neighbourhood trust was a powerful predictor of self-rated health. In line with this finding, an earlier study highlighted the importance of neighbourhood trust in the self-rated health [41]. They discovered that those who have a high level of neighbourhood trust are more likely to have good self-rated health. However, building neighbourhood trust can be difficult for non-regular employees. Building trust in neighbours is difficult due to the frequent change in residence and job, as trust is time dependent. Furthermore, a study conducted in the United States concluded that a high level of neighbourhood trust has a protective effect on the development of major depression and thus has a strong influence on how individuals perceive their health [9]. This implies that non-regular employees have limited opportunities and resources to tackle their stressful situations, but neighbourhood trust could help to ease it.

The results of the study showed that low social capital was linked to poor self-rated health among regular employees. However, unlike the other employment conditions, variables such as smoking status, occupation, and physical activity were influential in the last model. Despite the advantage of a secure job and a constant source of income, regular employees are severely affected by inflexibility, limited task control, and being cut off from their social environment due to their employment conditions. This leads to stress at work and most of them resort to smoking and drinking, which has serious implications for their health [42]. As indicated in the findings, the regular employees’ group has the lowest proportion of individuals with high social capital and therefore managing stress and depression using social capital might be challenging. As observed in non-regular employees, all five indicators of social capital were associated with self-rated health among the regular employees. This observation is in line with earlier studies where social capital, such as general trust, social participation, and frequency of talking with neighbours, was positively associated with self-rated health [43].

The resource embedded in an individual’s social network is known as social capital. Resources take different forms depending on the group members in an individual’s social network. Emotional support, for example, is typically provided by family members, close friends, and members of a close-knit neighbourhood referred to as the primary members of the social network. Secondary members of the social network are connections from professional life, political organisations, and religious organisations [37]. Although social capital from these sources is important in building high individual social capital, the social capital of the main members is the most important. This is reflected in the amount of time spent together, the emotional intensity of the relationship, the intimacy of mutual disclosure, and the reciprocity of the services provided [44]. This study demonstrates that the type of employment influences these key characteristics. In other words, the nature of employment may influence the affiliation of neighbours to a person’s primary social network. As a result, precariously employed people in the non-regular group may have fewer members in the primary social network group because they are unable to establish strong social ties or social capital with both work colleagues and neighbours over time. This is because they are constantly changing their place of employment and residence [45].

Accumulating high levels of social capital could improve the self-rated health of workers through a conscious attempt to carry out various meaningful community activities that improve emotional exchanges between neighbours, increase community participation among residents, and create a pleasant environment for people to relieve the pressures of life. Appropriate policies and interventions that create this pathway could be implemented to this end to improve the overall health of workers.

This study is one of the few to examine the relationship between social capital and self-rated health among the different employment types. Nevertheless, the major limitation of this study is that analysis of the cause-and-effect relationship between social capital and self-rated health was not done due to the study’s cross-sectional design. Additionally, the study did not consider the interaction effect of social capital and employment types. A longitudinal study would be necessary to investigate the interaction effect between these two variables on self-rated health. In addition, the data used in this study was collected in 2013. Nonetheless, the findings of the study are still relevant in today’s discussions.

## 5. Conclusions

The results of this study demonstrated that there is a significant positive relationship between social capital and self-rated health among workers. However, the self-rated health of the self-employees was highly related to the feeling of fellowship, social support, and cooperation; whereas, among the regular and non-regular employees, all the five measures of social capital influenced the association with self-rated health. This suggests that social capital acts as an unequal health resource for different workers. Therefore, when implementing interventions and policies to improve the health of workers based on their social capital, different approaches should be used for the different employment types.

## Figures and Tables

**Table 1:** Demographic characteristics of respondents according to employment type.

		Regular Employees	Non-Regular Employees	Self-Employed	
		(*n* = 2574)	(*n* = 1641)	(*n* = 701)	
Characteristics		*n* (%)	*n* (%)	*n* (%)	*p*-Value
Social capital					
	Low	132 (5.2)	96 (5.9)	32 (4.6)	<0.001
	Medium	1071 (42.0)	615 (37.8)	202 (29.1)	
	High	1346 (52.8)	914 (56.2)	461 (66.3)	
Gender					
	Men	1689 (66.8)	411 (25.4)	406 (58.7)	<0.001
	Women	840 (33.2)	1208 (74.6)	286 (41.3)	
Age (Years)					
	20–24	133 (5.2)	84 (5.1)	1 (0.1)	<0.001
	25–34	492 (19.1)	220 (13.4)	34 (4.9)	
	35–44	697 (27.1)	376 (22.9)	136 (19.4)	
	45–54	744 (28.9)	407 (24.8)	197 (28.1)	
	55–64	508 (19.7)	554 (33.8)	333 (47.5)	
Educational level					
	Low	1402 (55.0)	1021 (62.8)	415 (59.6)	<0.001
	Medium	544 (21.3)	397 (24.4)	148 (21.3)	
	High	605 (23.7)	209 (12.8)	133 (19.1)	
Occupation					
	Blue-collar	1083 (42.5)	493 (31.2)	319 (46.6)	<0.001
	White-collar	948 (37.2)	688 (43.6)	231 (33.7)	
	Others	519 (20.4)	397 (25.2)	135 (19.7)	
Annual Household Income (Million Yen)					
	<2	32 (1.6)	148 (12.1)	49 (9.4)	<0.001
	2–3.99	281 (14.0)	311 (25.5)	129 (24.6)	
	4–5.99	534 (26.5)	346 (28.3)	143 (27.3)	
	6–7.99	501 (24.9)	208 (17.0)	82 (15.6)	
	>7.99	666 (33.1)	209 (17.1)	121 (23.1)	
Marital Status					
	Never married	589 (23.0)	267 (16.4)	74 (10.6)	<0.001
	Currently married	1805 (70.4)	1214 (74.5)	581 (83.1)	
	Previously married	170 (6.6)	149 (9.1)	44 (6.3)	
Physical Activity					
	Inactive	2325 (91.3)	1469 (90.6)	612 (89.0)	0.169
	Active	222 (8.7)	153 (9.4)	76 (11.0)	
Smoking status					
	Currently smokes	770 (30.0)	293 (17.9)	180 (25.9)	<0.001
	Smoked before	702 (27.3)	372 (22.8)	248 (35.7)	
	Never smoked	1096 (42.7)	970 (59.3)	267 (38.4)	
Non-communicable disease					
	Absent	1263 (52.9)	779 (51.7)	308 (47.4)	0.046
	Present	1126 (47.1)	728 (48.3)	342 (52.6)	
BMI (kg/m^2^)					
	Underweight	173 (6.9)	204 (12.9)	39 (5.6)	<0.001
	Normal	1714 (68.6)	1136 (71.6)	465 (67.3)	
	Overweight	513 (20.5)	210 (13.2)	160 (23.2)	
	Obese	97 (3.9)	37 (2.3)	27 (3.9)	
Self-rated health					
	Poor	559 (21.8)	368 (22.5)	144 (20.5)	0.579
	Good	2010 (78.2)	1269 (77.5)	557 (79.5)	

**Table 2:** Characteristics distribution of respondents among social capital groups based on their employment type.

		Regular Employees			Non-Regular Employees			Self-Employed		
		Social Capital								
		Low	Medium	High	Low	Medium	High	Low	Medium	High
		(*n* = 132)	(*n* = 1071)	(*n* = 1346)	(*n* = 96)	(*n* = 615)	(*n* = 914)	(*n* = 32)	(*n* = 202)	(*n* = 461)
Characteristics		*n* (%)	*n* (%)	*n* (%)	*n* (%)	*n* (%)	*n* (%)	*n* (%)	*n* (%)	*n* (%)
Self-rated health										
	Poor	46 (34.8)	255 (23.9)	251 (18.7)	39 (40.6)	147 (24.0)	174 (19.1)	10 (31.2)	47 (23.3)	86 (18.7)
	Good	86 (65.2)	813 (76.1)	1093 (81.3)	57 (59.4)	465 (76.0)	739 (80.9)	22 (68.8)	155 (76.7)	375 (81.3)
Gender										
	Men	79 (61.2)	670 (63.7)	922 (69.6)	24 (25.0)	161 (26.6)	219 (24.3)	20 (62.5)	125 (63.1)	255 (55.9)
	Women	50 (38.8)	381 (36.3)	402 (30.4)	72 (75.0)	445 (73.4)	682 (75.7)	12 (37.5)	73 (36.9)	201 (44.1)
Age (Years)										
	20–24	15 (11.4)	79 (7.4)	34 (2.5)	17 (17.7)	45 (7.3)	21 (2.3)	0 (0.0)	1 (0.5)	0 (0.0)
	25–34	40 (30.3)	272 (25.4)	175 (13.0)	21 (21.9)	118 (19.2)	80 (8.8)	0 (0.0)	15 (7.4)	18 (3.9)
	35–44	36 (27.3)	323 (30.2)	333 (24.7)	21 (21.9)	143 (23.3)	211 (23.1)	7 (21.9)	56 (27.7)	73 (15.8)
	45–54	21 (15.9)	275 (25.7)	442 (32.8)	22 (22.9)	141 (22.9)	241 (26.4)	9 (28.1)	53 (26.2)	133 (28.9)
	55–64	20 (15.2)	122 (11.4)	362 (26.9)	15 (15.6)	168 (27.3)	361 (39.5)	16 (50.0)	77 (38.1)	237 (51.4)
Educational level										
	Low	69 (52.7)	557 (52.7)	760 (56.8)	58 (61.1)	366 (60.0)	588 (64.8)	22 (68.8)	118 (59.3)	271 (59.0)
	Medium	36 (27.5)	238 (22.5)	265 (19.8)	19 (20.0)	157 (25.7)	218 (24.0)	3 (9.4)	42 (21.1)	103 (22.4)
	High	26 (19.8)	261 (24.7)	314 (23.5)	18 (18.9)	87 (14.3)	101 (11.1)	7 (21.9)	39 (19.6)	85 (18.5)
Occupation										
	Blue-collar	46 (35.1)	454 (42.8)	570 (42.7)	30 (34.5)	197 (33.4)	261 (29.3)	13 (41.9)	88 (44.4)	216 (48.0)
	White-collar	61 (46.6)	411 (38.7)	473 (35.4)	39 (44.8)	244 (41.4)	404 (45.4)	10 (32.3)	65 (32.8)	153 (34.0)
	Others	24 (18.3)	196 (18.5)	293 (21.9)	18 (20.7)	149 (25.3)	225 (25.3)	8 (25.8)	45 (22.7)	81 (18.0)
Annual Household Income (Million Yen)										
	<2	2 (2.3)	13 (1.6)	17 (1.5)	20 (30.3)	57 (13.0)	67 (9.5)	4 (18.2)	14 (9.1)	30 (8.7)
	2–3.99	18 (20.5)	138 (17.2)	122 (10.9)	19 (28.8)	123 (28.0)	166 (23.4)	5 (22.7)	38 (24.7)	86 (24.9)
	4–5.99	25 (28.4)	216 (26.9)	293 (26.3)	6 (9.1)	129 (29.4)	210 (29.7)	2 (9.1)	43 (27.9)	97 (28.0)
	6–7.99	19 (21.6)	193 (24.1)	286 (25.6)	13 (19.7)	64 (14.6)	130 (18.4)	2 (9.1)	22 (14.3)	58 (16.8)
	>7.99	24 (27.3)	242 (30.2)	398 (35.7)	8 (12.1)	66 (15.0)	135 (19.1)	9 (40.9)	37 (24.0)	75 (21.7)
Marital Status										
	Never married	56 (42.4)	320 (30.0)	202 (15.1)	35 (36.5)	151 (24.7)	77 (8.5)	6 (18.8)	23 (11.5)	45 (9.8)
	Currently married	62 (47.0)	668 (62.7)	1062 (79.1)	47 (49.0)	392 (64.1)	768 (84.8)	25 (78.1)	161 (80.5)	393 (85.2)
	Previously married	14 (10.6)	78 (7.3)	78 (5.8)	14 (14.6)	69 (11.3)	61 (6.7)	1 (3.1)	16 (8.0)	23 (5.0)
Physical Activity										
	Inactive	123 (96.1)	979 (92.2)	1201 (90.0)	91 (95.8)	548 (90.3)	820 (90.5)	27 (87.1)	184 (91.5)	397 (87.8)
	Active	5 (3.9)	83 (7.8)	134 (10.0)	4 (4.2)	59 (9.7)	86 (9.5)	4 (12.9)	17 (8.5)	55 (12.2)
Smoking status										
	Currently smokes	38 (29.0)	350 (32.8)	374 (27.8)	28 (29.5)	119 (19.4)	141 (15.5)	9 (28.1)	59 (29.4)	110 (24.1)
	Smoked before	33 (25.2)	230 (21.6)	429 (31.9)	17 (17.9)	138 (22.5)	212 (23.3)	11 (34.4)	80 (39.8)	153 (33.6)
	Never smoked	60 (45.8)	487 (45.6)	542 (40.3)	50 (52.6)	357 (58.1)	558 (61.3)	12 (37.5)	62 (30.8)	193 (42.3)
Non-communicable disease										
	Absent	63 (51.6)	576 (58.1)	608 (48.5)	47 (52.2)	302 (53.1)	425 (50.8)	16 (51.6)	102 (55.7)	189 (43.9)
	Present	59 (48.4)	416 (41.9)	646 (51.5)	43 (47.8)	267 (46.9)	411 (49.2)	15 (48.4)	81 (44.3)	242 (56.1)
BMI (kg/m^2^)										
	Underweight	13 (10.1)	92 (8.9)	66 (5.0)	19 (20.2)	91 (15.3)	94 (10.6)	4 (12.5)	7 (3.5)	28 (6.2)
	Normal	80 (62.0)	707 (68.5)	913 (69.4)	57 (60.6)	406 (68.5)	664 (74.9)	16 (50.0)	146 (73.0)	299 (66.0)
	Overweight	28 (21.7)	196 (19.0)	285 (21.7)	16 (17.0)	76 (12.8)	115 (13.0)	12 (37.5)	37 (18.5)	110 (24.3)
	Obese	8 (6.2)	37 (3.6)	51 (3.9)	2 (2.1)	20 (3.4)	14 (1.6)	0 (0.0)	10 (5.0)	16 (3.5)

**Table 3:** Prevalence ratios of social capital for poor self-rated health respondents.

	Crude Model			Model 1 ^a^			Model 2 ^b^			Model 3 ^c^		
	OR	95% C.I. for OR		OR	95% C.I. for OR		OR	95% C.I. for OR		OR	95% C.I. for OR	
		Lower	Upper		Lower	Upper		Lower	Upper		Lower	Upper
Social capital												
Regular Employees												
Low	Reference			Reference			Reference			Reference		
Medium	0.589	0.401	0.865	0.554	0.376	0.816	0.551	0.372	0.815	0.583	0.391	0.870
High	0.433	0.295	0.635	0.372	0.251	0.551	0.369	0.248	0.550	0.393	0.262	0.590
Non-Regular Employees												
Low	Reference			Reference			Reference			Reference		
Medium	0.481	0.308	0.751	0.433	0.274	0.686	0.471	0.296	0.752	0.466	0.289	0.751
High	0.346	0.223	0.537	0.306	0.193	0.484	0.341	0.213	0.546	0.339	0.209	0.550
Self-Employed												
Low	Reference			Reference			Reference			Reference		
Medium	0.659	0.291	1.489	0.699	0.307	1.593	0.838	0.361	1.949	0.727	0.301	1.756
High	0.506	0.231	1.108	0.507	0.230	1.116	0.598	0.266	1.344	0.490	0.211	1.137

C.I., confidence interval; OR, Odds ratio. ^a^ Adjusted for Gender, Age (Years). ^b^ Adjusted for Gender, Age (Years), Annual Household Income (Million Yen), Education Attainment, Job, and Marital Status. ^c^ Adjusted for Gender, Age (Years), Annual Household Income (Million Yen), Education Attainment, Job, Smoking status, Physical Activeness, Non-Communicable Disease, and Body Mass Index (BMI).

**Table 4:** Prevalence ratios of each social capital dimension for poor self-rated health respondents.

		Crude Model			Model 1 ^a^			Model 2 ^b^			Model 3 ^c^		
		OR	95% C.I. for OR		OR	95% C.I. for OR		OR	95% C.I. for OR		OR	95% C.I. for OR	
			Lower	Upper		Lower	Upper		Lower	Upper		Lower	Upper
Feeling of Fellowship													
Regular Employees													
Low	Reference				Reference			Reference			Reference		
Medium	0.753		0.559	1.015	0.717	0.530	0.968	0.705	0.520	0.954	0.728	0.535	0.991
High	0.650		0.496	0.851	0.569	0.430	0.753	0.567	0.427	0.754	0.590	0.441	0.788
Non-Regular Employees													
Low	Reference				Reference			Reference			Reference		
Medium	0.687		0.471	1.002	0.655	0.447	0.959	0.705	0.478	1.040	0.718	0.482	1.069
High	0.503		0.360	0.703	0.476	0.337	0.672	0.513	0.360	0.731	0.525	0.366	0.755
Self-Employed													
Low	Reference				Reference			Reference			Reference		
Medium	0.671		0.528	0.853	0.657	0.516	0.836	0.660	0.518	0.841	0.667	0.522	0.853
High	0.616		0.496	0.766	0.563	0.450	0.704	0.559	0.446	0.701	0.567	0.451	0.714
Social support													
Regular Employees													
Low	Reference				Reference			Reference			Reference		
Medium	0.671		0.528	0.853	0.657	0.516	0.836	0.660	0.518	0.841	0.667	0.522	0.853
High	0.616		0.496	0.766	0.563	0.450	0.704	0.559	0.446	0.701	0.567	0.451	0.714
Non-Regular Employees													
Low	Reference				Reference			Reference			Reference		
Medium	0.694		0.507	0.950	0.679	0.495	0.933	0.716	0.519	0.986	0.727	0.525	1.007
High	0.609		0.468	0.794	0.604	0.460	0.792	0.630	0.476	0.833	0.636	0.478	0.846
Self-Employed													
Low	Reference				Reference			Reference			Reference		
Medium	0.487		0.285	0.833	0.485	0.282	0.832	0.453	0.261	0.787	0.450	0.253	0.799
High	0.533		0.347	0.818	0.531	0.343	0.821	0.530	0.341	0.826	0.485	0.306	0.768
Cooperation													
Regular Employees													
Low	Reference				Reference			Reference			Reference		
Medium	0.858		0.679	1.084	0.790	0.622	1.003	0.782	0.614	0.995	0.802	0.628	1.024
High	0.659		0.524	0.828	0.566	0.444	0.721	0.558	0.436	0.715	0.568	0.442	0.730
Non-Regular Employees													
Low	Reference				Reference			Reference			Reference		
Medium	1.068		0.799	1.427	1.033	0.770	1.386	1.079	0.800	1.455	1.081	0.797	1.467
High	0.687		0.515	0.917	0.657	0.487	0.886	0.701	0.516	0.953	0.688	0.504	0.941
Self-Employed													
Low	Reference				Reference			Reference			Reference		
Medium	0.554		0.324	0.945	0.523	0.304	0.899	0.524	0.301	0.914	0.506	0.283	0.906
High	0.615		0.389	0.971	0.537	0.335	0.861	0.523	0.320	0.853	0.479	0.287	0.799
Reciprocity Norm													
Regular Employees													
Low	Reference				Reference			Reference			Reference		
Medium	0.749		0.550	1.021	0.724	0.530	0.988	0.693	0.506	0.950	0.686	0.498	0.944
High	0.631		0.468	0.849	0.602	0.446	0.813	0.594	0.439	0.804	0.600	0.441	0.816
Non-Regular Employees													
Low	Reference				Reference			Reference			Reference		
Medium	0.829		0.581	1.184	0.811	0.565	1.162	0.850	0.590	1.225	0.877	0.605	1.272
High	0.528		0.375	0.743	0.518	0.367	0.733	0.545	0.383	0.775	0.562	0.393	0.805
Self-Employed													
Low	Reference				Reference			Reference			Reference		
Medium	0.876		0.483	1.586	0.879	0.483	1.602	0.850	0.460	1.572	0.774	0.409	1.464
High	0.613		0.351	1.069	0.613	0.349	1.076	0.602	0.339	1.069	0.560	0.309	1.017
Perceived Neighbourhood Trust													
Regular Employees													
Low	Reference				Reference			Reference			Reference		
Medium	0.657		0.511	0.845	0.639	0.496	0.823	0.639	0.495	0.824	0.653	0.504	0.846
High	0.470		0.362	0.609	0.437	0.336	0.570	0.440	0.337	0.575	0.454	0.346	0.596
Non-Regular Employees													
Low	Reference				Reference			Reference			Reference		
Medium	0.597		0.444	0.802	0.581	0.431	0.783	0.620	0.458	0.840	0.647	0.475	0.881
High	0.446		0.328	0.606	0.435	0.319	0.595	0.459	0.333	0.631	0.468	0.339	0.648
Self-Employed													
Low	Reference				Reference			Reference			Reference		
Medium	0.889		0.530	1.492	0.844	0.500	1.424	0.826	0.484	1.412	0.887	0.505	1.559
High	0.559		0.333	0.939	0.526	0.311	0.890	0.534	0.313	0.910	0.577	0.330	1.009

C.I., confidence interval; OR, Odds ratio. ^a^ Adjusted for Gender, Age (Years). ^b^ Adjusted for Gender, Age (Years), Annual Household Income (Million Yen), Education Attainment, Job, and Marital Status. ^c^ Adjusted for Gender, Age (Years), Annual Household Income (Million Yen), Education Attainment, Job, Smoking status, Physical Activeness, Non-Communicable Disease, and BMI.

## Data Availability

The data presented is available upon request from the corresponding author. The data are not publicly available since they contain information that could compromise the privacy of research participants.
